# SLFN11: a pan-cancer biomarker for DNA-targeted drugs sensitivity and therapeutic strategy guidance

**DOI:** 10.3389/fonc.2025.1582738

**Published:** 2025-07-22

**Authors:** Kunzhong Zhou, Yuewen Li, Weifang Wang, Yilin Chen, Bingyan Qian, Yiteng Liang, Hongmei Li, Ruiting Xu, Li Zhuang

**Affiliations:** Department of Rehabilitation and Palliative Medicine, The Third Affiliated Hospital of Kunming Medical University, Yunnan Cancer Hospital, Peking University Cancer Hospital Yunnan, Kunming, China

**Keywords:** Schlafen11 (SLFN11), DNA damaging agents, PARP inhibitors, pan-cancer, DNA damage repair mechanisms, epigenetics, biomarkers

## Abstract

Therapeutic responses to identical chemotherapy regimens often vary significantly among patients with the same type of cancer, underscoring the need for additional biomarkers to identify individuals most likely to benefit from specific treatments. The expression of SLFN11 (Schlafen11) has been identified as a potential biomarker for predicting patient responses to DNA-damaging agents and PARP inhibitors, as it irreversibly blocks DNA replication under replication stress, thereby increasing the sensitivity of cancer cells to various DNA-damaging agents and PARP inhibitors. Preclinical and clinical trial data suggest that SLFN11 can predict therapeutic responses to multiple DNA- targeted drugs, including platinum-based agents, topoisomerase I/II inhibitors, DNA synthesis inhibitors, and PARP inhibitors. Leveraging the expression status of SLFN11 or modulating its expression offers exciting possibilities for clinical applications. In this review, we summarize the structure and function of SLFN11, as well as its progress as a biomarker across various cancer types. We also review the regulation of SLFN11 expression, its dynamic expression patterns, and potential strategies for combination therapies to enhance efficacy based on SLFN11 status. Furthermore, we discuss the potential of SLFN11 expression status in overcoming resistance to DNA-damaging drugs, optimizing treatment strategies, and advancing precision cancer therapy.

## 1 Introduction

Cancer continues to face challenges such as recurrence, drug side effects, drug resistance, and individual variations in treatment efficacy (1). The economic burden of 29 types of cancer across 204 countries and regions is projected to reach $25.2 trillion from 2020 to 2050 (2). The six hallmarks of cancer include sustaining proliferative signaling, evading growth suppressors, resisting cell death, enabling replicative immortality, inducing angiogenesis, and activating invasion and metastasis, all of which are underpinned by genomic instability (3). For decades, chemotherapy and radiation therapy have been the cornerstones of cancer treatment, but sensitivities vary in unselected patients. Recently, more and more studies have found that the expression status of SLFN11 is associated with the chemotherapy sensitivity of tumor patients. These therapeutic drugs include platinum, topoisomerase I/II inhibitors, DNA damaging agents, and PARP inhibitors, which we collectively refer to as DNA-targeted drugs in this article. Small molecule inhibitors targeting the DNA damage response (DDR) have garnered significant interest. A prime example is PARP inhibitors, particularly in ovarian cancer with BRCA1/2 mutations, which are the most common cause of homologous recombination repair deficiency (HRD). Since these cells rely on PARP1/2 for single-strand repair, the use of PARPi leads to a “synthetic lethality” effect. In addition to BRCA gene mutations predicting the efficacy of PARP inhibitors, SLFN11 can also predict the therapeutic sensitivity of PARP inhibitors. SLFN11 has been recognized as a biomarker predictive of response to various DNA-damaging agents and PARPi across multiple cancer types, including gastric cancer (4), esophageal cancer (5), small cell lung cancer (6–9), breast cancer (10), ovarian cancer (11, 12), prostate cancer (13), Ewing sarcoma (14), glioblastoma (15), head and neck cancer (16), colorectal cancer (17, 18), clear cell renal cell carcinoma (19) and hepatocellular carcinoma (20). These findings provide a foundation for the clinical application of SLFN11 as a biomarker. In this article, we review the structure and function of SLFN11. We also review the progress in research on SLFN11 as a biomarker in various cancers, with a focus on SCLC. Additionally, we review the regulation and dynamic changes of SLFN11 expression and potential strategies for combination therapy based on its expression status. Finally, we discuss the potential of SLFN11 in overcoming drug resistance, optimizing treatment strategies, and advancing precision cancer therapy.

## 2 The structure and function of SLFN11

The SLFN gene family was first described in 1998 as a growth-regulating gene family influencing thymocyte development (21). The murine SLFN family comprises 10 members (SLFN1, 1L, 2, 3, 4, 5, 8, 9, 10, and 14), while the human SLFN family consists of 6 members (SLFN5, 11, 12, 12L, 13, and 14) (22). All human SLFN genes contain an SLFN box, a domain not found in other proteins, whose specific function remains to be elucidated. Except for the lack of a helicase domain in SLFN12, the remaining human SLFN proteins contain a helicase domain at the C - terminus (11).

Over the past decade, SLFN11 has been extensively studied for its relevance to cancer therapy. The SLFN11 gene is located on human chromosome 17 and encodes a protein consisting of 901 amino acid residues containing three major domains (11, 23): an N-terminal endonuclease domain (residues 1–353), an intermediate linker domain (residues 354–576), and a C-terminal domain (residues 577–901). The N-terminal domain is the critical domain of SLFN11, possessing endoribonuclease activity (22). Under DNA damage induction, SLFN11 mediates the cleavage of type II tRNAs (notably tRNA-Leu-TAA) through its N-terminal endoribonuclease activity, targeting their long variable loops. This degradation selectively disrupts the translation of DNA damage response and repair genes such as ATR and ATM, whose transcripts are enriched in TTA codons (Leu) that depend on the low-abundance tRNA-Leu-TAA for efficient protein synthesis (24). ATR (Ataxia Telangiectasia and Rad3-related) and ATM (Ataxia Telangiectasia Mutated) protein kinases are members of the phosphatidylinositol 3-kinase (PI3K)-related kinase (PIKK) protein family and play a key role in DNA damage response (25). ATR is mainly involved in the response to replication stress and maintaining replication fork stability. ATM is mainly involved in DNA double-strand break repair, regulating cell cycle checkpoints, and promoting homologous recombination (HR) repair. In response to DNA damage, it inhibits protein translation by degrading specific tRNAs, promoting the sensitivity of cancer cells to DNA-damaging agents (22). Studies have shown that mouse Slfn8 and Slfn9 may partially compensate for the function of human SLFN11 (26), but phylogenetic and sequence alignment analysis showed that mouse SLFN8/9/10 are orthologous genes of human SLFN13 rather than SLFN11 (27). Studies have determined the crystal structure and function of the Sus scrofa (wild boar) SLFN11 N-terminal domain (NTD). sSLFN11-NTD is a clamp molecule and an efficient RNase that cleaves type I and II tRNA and rRNA, and preferentially cleaves type II tRNA (27). Cryo-EM structures reveal that SLFN11 interacts with tRNA through the positively charged groove formed by the N-terminal nuclease domain of its dimer. The structure captured the binding state of SLFN11 with tRNA-Leu (type II) and tRNA-Met (type I) and confirmed that both tRNAs were cleaved at specific sites 10 nucleotides away from the 3’ end (positions 76–77 for tRNA-Leu, positions 65–66 for tRNA-Met) (28). The phosphorylation sites S219 and T230 located in the N-terminal nuclease domain regulate tRNA recognition and ribonuclease activity. After phosphorylation, the negative charge repels the tRNA phosphate backbone, weakening the tRNA binding ability and resulting in a significant reduction in nuclease activity (28). The intermediate connecting domain of SLFN11 contains a conserved SWAVDL sequence, which is present in all SLFN family members (22). The C-terminal domain is homologous to superfamily I RNA/DNA helicases and contains a conserved Walker A/B motif (ATPase active site). Structural simulations show similarity to Dna2 helicase, suggesting involvement in chromatin remodeling (23, 29). Helicase activity is required for SLFN11-mediated chemosensitivity to DNA-damaging agents and replication fork degradation (30). The phosphorylation site S753 located in the C-terminal helicase domain acts as a conformational switch to regulate SLFN11 dimerization and nucleic acid binding ability. SLFN11 switches between monomer and dimer conformations through the phosphorylation state of S753. S753 phosphorylation induces a 140° rotation of the C-terminal helicase domain, destroying the ID helix and hydrophobic interactions to form a monomer conformation. S753 dephosphorylation is a key trigger for dimerization. Protein phosphatase 1 catalytic subunit γ (PPP1CC)-mediated S753 dephosphorylation relieves conformational inhibition and promotes dimer formation. The monomeric SLFN11 has the following characteristics: no DNA binding, weakened nuclease activity, binding to ATP but no hydrolysis, and maintaining a “closed” state. The dimeric SLFN11 has the following characteristics: binding to ssDNA/tRNA, high cleavage activity, and performing replication fork arrest and translation regulation (28). S753 phosphorylation acts as a “safety lock” to inhibit SLFN11 activity under non-stress conditions, preventing abnormal replication fork blockage or excessive tRNA cleavage. When DNA is damaged, PPP1CC-mediated S753 dephosphorylation can activate SLFN11 dimerization, enabling it to coordinate the execution of replication fork blockage and tRNA cleavage functions. Dimerization underlies SLFN11-dependent chemosensitivity. In addition, studies have found that the single-stranded DNA (ssDNA) binding site K652 in the SLFN11 protein is a direct binding site for ssDNA (31). K652 (lysine) is positively charged and can form electrostatic interactions with negatively charged ssDNA. When K652 mutates to negatively charged glutamic acid (K652E) or aspartic acid (K652D), SLFN11 loses its ssDNA binding ability and cannot be recruited to chromatin, losing its replication blocking function and completely losing its drug sensitivity. S753 dephosphorylation may change the protein conformation, expose the K652 site or optimize its interaction with ssDNA.

SLFN11 is recruited to DNA damage sites through direct binding with RPA, promoting the destabilization of the RPA-ssDNA complex, thereby inhibiting checkpoint maintenance and homologous recombination repair (23, 32). SLFN11 promotes the degradation of CDT1 in response to CPT by binding to DDB1 of CUL4^CDT2^ E3 ubiquitin ligase associated with replication forks, which irreversibly blocks replication and induces cell death (33). The SLFN11 protein enhances chromatin accessibility across the genome, particularly in response to replication stress induced by DNA-targeting drugs, with this increase being most pronounced in active gene promoter regions (34). Additionally, it responds to replication stress by regulating immediate early genes (such as JUN, FOS) and cell cycle arrest genes (such as CDKN1A), with this function of SLFN11 dependent on its ATPase and C-terminal helicase activities. In response to replication stress induced by camptothecin or the CHK1 inhibitor Prexasertib, SLFN11 is recruited to stressed replication forks, blocking replication by altering chromatin structure (30). In immune responses, the expression of SLFN11 enhances the effect of the IFNγ signaling pathway, making tumor cells more sensitive to cytotoxic T cells (35).

## 3 The significance of SLFN11 in various cancers

SLFN11 is a very important and widely recognized biomarker for predicting sensitivity to multiple DNA-targeted drugs. People have made great progress in this area. In this review, we summarized the role, mechanism and clinical significance of SLFN11 in gastric cancer, esophageal cancer, small cell lung cancer, breast cancer, ovarian cancer, prostate cancer, Ewing’s sarcoma, glioblastoma, head and neck cancer, colorectal cancer, renal cancer, hepatocellular carcinoma and other cancers (**Tables 1**, **2**).

**Table 1:** Studies that evaluated SLFN11 as a prognostic or predictive biomarker in cancer patients.

Evidence level	Type of cancer	n of patients	Drugs	Conclusions	Ref.
Retrospective study	Esophageal Squamous Cell Carcinoma	73	low-dose nedaplatin + 5-fluorouracil with concurrent radiation	Tumors with high SLFN11 H-score(≥ 51) were associated with longer PFI (p = 0.013).	(36)
Prospective Phase II study	Recurrent small cell lung cancer	104	Temozolomide +veliparib or placebo	Temozolomide + veliparib elicited longer PFS (5.7 v 3.6 months; p = 0.009) and OS (12.2 v 7.5 months; p = 0.014) in patients with SLFN11+ tumors vs. SLFN11- tumors(H score cutoff ≥1 defined SLFN11 positive).	(37)
Prospective I/II-phase study	Recurrent small cell lung cancer	21	valemetostat (DS-3201b) combination with irinotecan	Combination EZH1/2 inhibitor valemetostat and irinotecan was not tolerated but demonstrated efficacy in recurrent SCLC	(38)
Prospective Phase II study	SLFN11-positive ES-SCLC	106	atezolizumab (A) versus atezolizumab plus talazoparib (AT)	PFS was improved with AT versus A (2.9 v 2.4 months; p = 0.019); OS was not different between groups (p = 0.47).	(39)
Observational study	Non-small cell lung cancer	22	Platinum-based chemotherapy	SLFN11 promoter methylation was associated with poor PFS (p = 0.031).	(6)
Observational study	Breast Cancer	32	chemotherapy (Not specified)	High SLFN11 mRNA levels were associated with better OS (p = 0.017).	(10)
Retrospective study	high-grade serous ovarian cancer	27	platinum-based chemotherapy	Tumors with high SLFN11 H-score(“high” if H-score > 60) were associated with longer PFI (p = 0.004).	(12)
Retrospective study	Ovarian Cancer	110	Cisplatin-based chemotherapy	High SLFN11 mRNA levels were associated with better OS (p = 0.016).	(40)
Observational study	Ovarian Cancer	41	Cisplatin or carboplatin	SLFN11 promoter rmethylation was associated with shorter (OS) (p = 0.006) and PFS (p = 0.003).	(6)
Retrospective study	Castration-Resistant Prostate Cancer	20	platinum-based chemotherapy	Longer rPFS was associated with SLFN11+ CTCs compared to those without (6.0 versus 2.2 months, p=0.002)	(41)
Retrospective study	Ewing Sarcoma	44	Not specified	Tumors with high SLFN11 mRNA levels were associated with longer RFS (p = 0.0046).	(14)
Retrospective study	Head and Neck Squamous Cell Carcinoma	161	Platinum (cisplatin or carboplatin)-based chemoradiotherapy	Tumors with SLFN11-positive(SLFN11 positive staining was defined as ≥15% staining of the tumor nuclei) were associated with longer PFS p < 0.001).	(16)
Retrospective study	Colorectal Cancer	128	Not specified	SLFN11 promoter methylation was prognostic of poor 5-year OS and 5-year RFS (p<0.05).	(40)
Retrospective study	Colorectal Cancer with KRAS exon 2 wild type	153	Adjuvant oxaliplatin-based chemotherapy	Tumors with high SLFN11 expression score(>4.5) were associated with longer OS (p = 0.048).	(18)
Retrospective study	Hepatocellular Carcinoma	182	Underwent curative hepatectomy	Tumors with high SLFN11(moderate or strong H-score) were associated with longer OS andRFS (p < 0.001).	(20)
Retrospective study	Bladder Cancer	50	Platinum-based chemotherapy	Tumors with SLFN11-positive (SLFN11 was considered positive when at least 5% of the tumor cells were stained)were associated with longer OS p < 0.012).	(42)

**Table 2:** Summary of the roles, mechanisms and the clinical significance of SLFN11 in different cancer types.

Cancer types	Expression characteristics of SLFN11	Functional mechanism	Clinical significance
Gastric cancer	High expression in tumor tissues	Promote cisplatin-induced S-phase arrest and apoptosis. The expression of SLFN11 is regulated by the methylation of the promoter region.	High expression is associated with the improvement of PFS. Methylation silencing leads to chemotherapy resistance.
Esophageal squamous cell carcinoma	Patients with high expression who receive chemotherapy/radiotherapy have a better prognosis(regulated by promoter methylation)	Inhibition of the ATM pathway enhances sensitivity to radiotherapy/chemotherapy. Methylation silencing is associated with poor tumor differentiation.	High expression is associated with the improvement of OS. Patients with SLFN11 deletion may be sensitive to ATM inhibitors (AZD0156).
Small cell lung cancer	High expression is associated with sensitivity to PARP inhibitors.	EZH2-mediated H3K27me3 deposition leads to downregulation of expression.	High expression is associated with the improvement of PFS/OS. Patients with SLFN11 positivity are sensitive to PARP inhibitors. EZH2 inhibitors can restore expression and overcome chemotherapy resistance.
Breast cancer	Patients with high expression who receive chemotherapy have a better prognosis.	–	High expression is associated with the improvement of OS. ATR inhibitors can reverse the drug resistance caused by low SLFN11 expression.
Ovarian cancer	Patients with high expression who receive chemotherapy have a better prognosis	–	High expression is associated with the improvement of OS. The expression status of SLFN11 can predict the efficacy of platinum-based drugs and PARP inhibitors.
Castration-resistant Prostate cancer	Patients with high expression levels have a better response to platinum-based chemotherapy.	–	High expression is associated with the improvement of radiographic progression-free survival (rPFS). The expression status of SLFN11 can predict the efficacy of platinum-based drugs.
Ewing Sarcoma	EWS-FLI1 transcriptional target genes, High expression in tumor cell	Impede replication repair and enhance the sensitivity to DNA - damaging drugs. Activate the AP-1 pathway to inhibit the oncogene c - Myc.	High expression is associated with the improvement of RFS, Patients with high expression of SLFN11 respond better to the combination of PARP inhibitors and topoisomerase inhibitors (such as SN - 38).
Glioblastoma	High expression promotes tumor progression.	Negatively regulate the NF-κB pathway and inhibit the expression of p21.	High expression is associated with the improvement of OS. SLFN11 deficiency inhibits tumor growth.
Head and Neck Squamous Cell Carcinoma	High expression of SLFN11 is associated with a longer PFS.	–	Patients with high expression of SLFN11 respond better to cisplatin-based chemoradiotherapy (CRT).
Clear Cell Renal Cell Carcinoma	High expression is associated with poor OS.	SLFN11 promotes the phosphorylation of the PI3K/AKT signaling pathway. SLFN11 is highly expressed in ccRCC tissues and cell lines, and is associated with a decreased methylation level.	Overexpression of SLFN11 is an independent prognostic factor for clear cell renal cell carcinoma.
Colorectal cancer	In patients receiving oxaliplatin adjuvant chemotherapy, high expression of SLFN11 is associated with a favorable prognosis in patients with wild - type KRAS exon 2.	Methylation leads to low expression. May interact with the KRAS mutation status.	Patients with high expression of SLFN11 and wild-type KRAS have a better prognosis after adjuvant oxaliplatin chemotherapy.
Hepatocellular carcinoma	Low expression is associated with poor prognosis.	Inhibiting the mTOR pathway through RPS4X. Regulating the TRIM21-RBM10 axis to enhance the response to immune checkpoint inhibitors (ICI)	Combination of CCL2/CCR2 inhibitors and PD - 1 inhibitors can improve the therapeutic efficacy in patients with low SLFN11 expression.
Leukemic cell lines	–	SLFN11 expression is regulated via the JAK, AKT and ERK, and ETS axis	–
Mesothelioma cell lines	–	–	The response of mesothelioma cells to PARP inhibitors is associated with high SLFN11 expression. When used in combination with temozolomide, it can increase the sensitivity of cells with low or no MGMT expression.

### 3.1 Gastric cancer

SLFN11 plays a complex role in gastric cancer. Its expression is epigenetically regulated (promoter region methylation), and high expression (especially protein level) is associated with better survival prognosis and is a powerful biomarker for predicting sensitivity to platinum-based chemotherapy. SLFN11 inhibits tumor growth and significantly enhances the efficacy of platinum drugs by promoting S phase arrest and apoptosis. At the same time, SLFN11 is deeply involved in the regulation of the tumor immune microenvironment and is positively correlated with the infiltration of multiple immune cells and the expression of immune checkpoint molecules, suggesting its potential immune regulatory function.

The TCGA database showed that the mRNA expression of SLFN11 in gastric cancer (STAD) was significantly higher than that in normal tissues. Analysis of the UALCAN database showed the mRNA level of SLFN11 was significantly positively correlated with lymph node metastasis, tumor stage and grade (43). The Kaplan-Meier Plotter showed that high expression of SLFN11 was not significantly correlated with patients’ overall survival (OS), and could not be used as a prognostic marker alone (unlike SLFN5/SLFN13) (43). However, a retrospective study evaluated the expression of SLFN11 in tumor cells using immunohistochemistry, and when >30% of tumor cells were stained, it was considered SLFN11 immunostaining positive. They used the median to divide patients into high SLFN11 group and low SLFN11 group. Kaplan-Meier analysis showed that the 5-year overall survival rates of 169 gastric cancer patients were 63% and 40% in the high SLFN11 group and the low SLFN11 group, respectively. The overall survival rate of the high SLFN11 group was significantly higher than that of the low SLFN11 group (HR, 0.5; 95% CI, 0.32-0.77; P = 0.0017). This difference was even more pronounced when analyzing patients who received either oxaliplatin or cisplatin(HR, 0.2; 95% CI, 0.06–0.51; *P* = 0.0009) *(*44). High expression of SLFN11 can be used as a predictive biomarker for gastric cancer patients receiving platinum-based chemotherapy (44).

GSEA functional enrichment analysis showed that SLFN11 was mainly involved in adaptive immune response and immune regulation (43). KEGG pathway analysis showed that SLFN11 was associated with inflammatory diseases (such as hepatitis, Epstein-Barr virus infection) and NF-κB signaling pathway (43). TIMER/TCGA database analysis showed that SLFN11 expression was positively correlated with the infiltration level of multiple immune cells, including CD8^+^ T cells, CD4^+^ T cells, macrophages (main associated cells), dendritic cells (DCs) (main associated cells), and neutrophils (43). TISIDB database shows that SLFN11 is positively correlated with NK cell, Th17 cell, and Treg cell infiltration (43). SLFN11 expression was significantly positively correlated with multiple immune checkpoint molecules, including CD160, CD244, CD247, CTLA4, LAG3, PDCD1, PDCD1LG2, TIGIT, and HAVCR2 (43).

At the epigenetic level, SLFN11 is frequently methylated in gastric cancer and its expression is regulated by promoter region methylation (4). Compared to normal gastric mucosal tissues, SLFN11 gene methylation is more prevalent in gastric cancer tissues, and the methylation rate of SLFN11 was significantly higher in tumors with a diameter ≥5 cm than in tumors with a diameter <5 cm. The use of the demethylating agent 5-AZA can restore SLFN11 expression (4). Restoring SLFN11 expression significantly inhibits the proliferative capacity of gastric cancer cells (such as SNU16 and MGC803). Studies using a mouse xenograft model have shown that the re-expression of SLFN11 significantly reduces tumor weight and volume. SLFN11 can enhance the sensitivity of gastric cancer cells to cisplatin by promoting cisplatin-induced S-phase arrest and apoptosis (4).

### 3.2 Esophageal cancer

SLFN11 is a key biomarker for the efficacy of chemoradiotherapy in ESCC. Its high expression improves platinum and radiotherapy sensitivity by regulating the DNA damage repair pathway (inhibiting ATM and activating ATR/NHEJ), and is dynamically regulated by epigenetic methylation. Targeting the ATM pathway in SLFN11-deficient tumors (such as AZD0156) has therapeutic potential.

In ESCC patients with definitive chemoradiotherapy (dCRT), those with high expression of SLFN11(H-score ≥ 51 was defined as high SLFN11 expression) exhibited significantly better prognosis (*p* = 0.013), particularly notable in stage II and III patients (*p* = 0.004) (36). This prognostic improvement is primarily attributed to the heightened sensitivity of SLFN11-high tumors to nedaplatin and radiotherapy, rather than to 5-fluorouracil (36). Using a low-dose cisplatin-induced DNA damage model, we found that SLFN11 was able to activate non-homologous end joining and ATR/CHK1 signaling pathways, while inhibiting the ATM/CHK2 signaling pathway (5). Loss of SLFN11 promotes tumor cell proliferation by restoring ATM expression (5). Studies have shown that SLFN11-deficient ESCC cells are highly sensitive to the ATM inhibitor AZD0156.

At the epigenetic level, the expression of SLFN11 is regulated by promoter methylation, which is significantly associated with tumor differentiation and tumor size (5). There was a negative correlation between SLFN11 mRNA levels and methylation of CpG sites around the transcription start site (cg13341380, cg18108623, cg05224998, cg18608369, cg01348733, cg14380270, cg26573518, and cg05504685, all P < 0.05) (5). Cell experiments showed that high expression of SLFN11 can enhance the sensitivity of ESCC cells to cisplatin (5). In KYSE30 and KYSE450 cell lines, after restoring SLFN11 expression, the expression of ATM was significantly inhibited.

### 3.3 Small cell lung cancer

Small cell lung cancer (SCLC) is a highly aggressive malignancy characterized by rapid progression and early metastasis. Although initial responses to platinum-based chemotherapy combined with etoposide are often favorable, the majority of patients relapse due to the rapid development of drug resistance, highlighting an urgent need for predictive biomarkers and more effective targeted therapies.

Recent molecular profiling has stratified SCLC into four distinct subtypes—SCLC-A, SCLC-N, SCLC-P, and SCLC-I—based on the expression of lineage-defining transcription factors ASCL1, NEUROD1, and POU2F3 (45). While SCLC-A and SCLC-N exhibit neuroendocrine features, SCLC-P and SCLC-I display non-neuroendocrine characteristics. Importantly, each subtype exhibits differential therapeutic responses: SCLC-I responds well to immunotherapy (particularly when combined with chemotherapy) due to its high expression of inflammation-related and immune checkpoint genes; SCLC-P shows particular sensitivity to PARP inhibitors; SCLC-N demonstrates good response to Aurora kinase inhibitors; and SCLC-A exhibits sensitivity to BCL-2 inhibitors. Notably, high expression of SLFN11 in the SCLC-A subtype is associated with sensitivity to PARP inhibitors, while the SCLC-P subtype remains sensitive to PARP inhibitors even in the absence of high SLFN11 expression or low ATM expression. POU2F3 expression, similar to SLFN11 expression, may serve as a predictive biomarker for PARP inhibitor sensitivity (45, 46). In SCLC-A (ASCL1-driven) cell lines, SLFN11 expression showed a bimodal distribution (45): (1) high peak population: SLFN11 was highly expressed and sensitive to cisplatin/PARPi. (2) low peak population: SLFN11 was low expressed and significantly resistant.

SLFN11 has emerged as a pivotal biomarker of response to DNA-damaging agents, particularly PARP inhibitors and platinum compounds. High SLFN11 expression correlates with enhanced drug sensitivity, while low expression confers resistance. This has been consistently validated in: SCLC cell lines, where SLFN11 expression negatively correlates with talazoparib IC50 values (7). PDX models, where SLFN11-high tumors show better responses to talazoparib (8). SCLC xenograft models, showing stronger effects of PARP inhibitors combined with temozolomide in SLFN11-positive tumors (7, 8). Mechanistically, Murai et al. proposed that SLFN11 enhances the activity of PARP inhibitors by inhibiting DNA replication (7), while others suggested that SLFN11 creates a “BRCAness” state by inhibiting homologous recombination repair (RPA-dependent mechanism), making cancer cells sensitive to PARP inhibitors (8, 32). High levels of SLFN11 (protein/mRNA) are the strongest predictors of SCLC sensitivity to PARP inhibitors (e.g., talazoparib, olaparib) and cisplatin, a finding that was validated in PDX models and 51 SCLC cell lines. Notably, SLFN11 protein expression was significantly downregulated in SCLC cells treated with cisplatin or PARP inhibitors (confirmed by western blotting) (9). The combined expression of SLFN11, low ATM expression, and epithelial phenotype (high E-cadherin expression/low EMT score) can optimize the prediction of SCLC treatment response (9). In summary, SLFN11 is a key dynamic regulator of SCLC sensitivity to DNA-damaging drugs, and PARP1 and ETS family transcription factor EHF regulate SLFN11 expression: PARP1 knockdown reduces SLFN11, while EHF is positively correlated with SLFN11 in SCLC and regulates its expression (knockdown of EHF reduces SLFN11). Promoter methylation is also involved in regulation, but demethylation treatment failed to effectively upregulate SLFN11 (9).

Further studies have expanded the potential of SLFN11 in combination therapy. Studies have shown that the downregulation of SLFN11 observed in chemoresistant SCLC patient-derived xenograft (PDX) models can be reversed by targeted epigenetic intervention (47, 48). Mechanism 1: EZH2-mediated trimethylation of histone H3 at lysine 27 (H3K27me3): EZH2, the catalytic subunit of the PRC2 complex, inhibits SLFN11 expression by depositing the repressive histone mark H3K27me3 specifically on the SLFN11 gene body. EZH2 inhibitors effectively reverse this silencing by reducing H3K27me3 levels and restoring SLFN11 expression, thereby resensitizing resistant SCLC models to chemotherapeutic drugs (48). Mechanism 2: Promoter methylation and histone deacetylation: In small cell lung cancer (SCLC) cell lines, SLFN11 expression is often silenced by promoter hypermethylation, which is significantly negatively correlated with SLFN11 expression (49). The histone deacetylase inhibitor (HDACi) FK228 reactivates SLFN11 expression primarily by increasing activating histone acetylation marks (H3K9Ac, H3K27Ac) at the promoter (47). Notably, this reactivation is associated with a decrease in promoter DNA methylation (47), suggesting that there may be a crosstalk between histone modifications and DNA methylation, although HDACs themselves act on histones rather than directly on DNA. FK228-induced restoration of SLFN11 expression effectively enhances the anticancer efficacy of topotecan (47). The DNA-damaging agent lurbinectedin effectively inhibits the proliferation of human SCLC cell lines, particularly those with high SLFN11 expression, while the combination of ATR inhibitors with lurbinectedin exhibits synergistic effects in SCLC cell lines with low SLFN11 expression (50). The novel ATR inhibitor M1774 has been shown to reverse chemotherapy resistance in SLFN11-deficient cells (51). Clinical sample analysis revealed that the proportion of SLFN11-positive circulating tumor cells is lowest in SCLC patients undergoing platinum-based therapy (52). This implies that SLFN11 expression levels decrease during platinum-based treatment. Dynamic expression of SLFN11 in circulating tumor cells can be used as a liquid biomarker for small cell lung cancer, which can predict patient sensitivity to treatment (52).

Multiple clinical studies have explored the practical application of SLFN11 as a predictive biomarker. In patients with recurrent SCLC, SLFN11-positive tumors(H score cutoff ≥1 defined SLFN11 positive) exhibited better responses to the combination therapy of temozolomide and veliparib, with significantly prolonged PFS and OS (37). Furthermore, following first-line chemotherapy, the maintenance therapy with the PARP inhibitor talazoparib combined with the immune checkpoint inhibitor atezolizumab significantly improved PFS in SLFN11-positive patients, although it did not significantly extend OS (39). The EZH2-SLFN11 pathway is a potentially targetable driver of acquired chemotherapy resistance. A single-arm phase I/II clinical trial reported that the combination of the EZH1/2 inhibitor Valemetostat (DS-3201b) with irinotecan in patients with recurrent small cell lung cancer presented toxicity issues, but some patients showed clinical benefit. No significant correlation was observed between SLFN11/EZH2 expression and SCLC subtype with treatment response (38).

Research on SLFN11 has also extended to non-small cell lung cancer (NSCLC). *In vitro* silencing of SLFN11 gene expression increases resistance to cisplatin and carboplatin in lung cancer cell lines (6). Clinical sample analysis revealed that SLFN11 methylation is associated with shortened PFS and OS in lung adenocarcinoma patients receiving platinum-based chemotherapy (6). NSCLC circulating tumor cell-derived xenograft (CDX) models and cell lines with high SLFN11 protein expression were more sensitive to PARP inhibitors, and CDX models and cell lines with high SLFN11 protein expression exhibited stronger metastatic potential and potential SCLC histological transformation (53).NSCLC cell lines with low SLFN11 expression and high cMYC expression demonstrated higher sensitivity to combined AXL/ATR inhibition therapy (54).

### 3.4 Breast cancer

The expression status of SLFN11 provides dual value for the precision treatment of breast cancer: on the one hand, it is a powerful biomarker for predicting the patient’s response and prognosis to DNA-damaging chemotherapy (including traditional chemotherapy, pyrrolobenzodiazepine (PBD)-conjugated antibody-drug conjugates (ADCs), PARP inhibitors, TOP1 inhibitors, etc.), and can be used to guide treatment selection and patient stratification; on the other hand, for the drug resistance caused by low SLFN11 expression, the combination treatment strategy targeting the DNA damage response pathway (such as ATR, CHK1, WEE1, EZH2) shows significant reversal potential, providing a new direction for overcoming drug resistance.

Survival analysis of clinical samples showed that breast cancer patients with high SLFN11 expression who received chemotherapy (unspecified drug) had a significant OS advantage (10). The expression of SLFN11 is strictly regulated in breast cancer, and its promoter methylation is an important mechanism leading to the downregulation of its mRNA and protein expression (55). In cell line models, upregulation of SLFN11 expression using IFN-γ, the demethylating agent DAC, or CRISPR-UNISAM significantly enhanced the sensitivity of cells to multiple DNA damaging agents, including cisplatin, epirubicin, and olaparib (55). This association was further supported at the patient level and in models: breast cancer patients with high SLFN11 protein expression responded significantly better to standard chemotherapy with DNA damaging agents (DDAs), such as gemcitabine and cisplatin (56). Conversely, knockdown of SLFN11 expression in the MDA-MB-361 cell line resulted in resistance or significant reduction in sensitivity to SG3199 (free pyrrolobenzodiazepine(PBD)) and PBD-antibody drug conjugates (e.g., MEDI0641, trastuzumab-SG3249) (57). Importantly, combination therapy strategies, such as PBD-ADC combined with ATR inhibitors (AZD6738) or EZH2 inhibitors, can effectively restore the sensitivity of SLFN11 low-expressing or null cells to these drugs (57). In a xenograft (PDX) model of triple-negative breast cancer (TNBC), the combination of irinotecan (TOP1 inhibitor) and ATR inhibitor VE-822 significantly improved tumor growth inhibition and inhibited CHK1 phosphorylation in SLFN11-negative tumors, overcoming the limitations of single-drug therapy (58). For breast cancer patients with low SLFN11 expression, preclinical evidence (56) suggests that the combination of DDA (such as gemcitabine) and ATR/WEE1/CHK1 inhibitors (such as AZD6738) may be an effective treatment strategy to overcome their potential drug resistance.

### 3.5 Ovarian cancer

SLFN11 is a powerful prognostic and predictive biomarker in ovarian cancer (especially high-grade serous ovarian cancer, HGSOC). Its expression level affects patient survival, response to platinum/PARPi, and is associated with the immune microenvironment. Its mechanism of inhibiting DDR (especially the ATR pathway) provides a theoretical basis for the use of targeted drugs (such as ATR inhibitors) to treat tumors with low SLFN11 expression. Future studies should be committed to verifying the clinical application value of SLFN11 as a predictive marker to guide targeted therapies such as PARP inhibitors (especially in BRCA wild-type populations) and ATR inhibitors.

High expression (mRNA expression above the median) of SLFN11 is significantly associated with longer overall survival (OS) and better efficacy of platinum-based drugs in ovarian cancer patients receiving cisplatin chemotherapy (10, 11). In HGSOC, high SLFN11 expression (“high” if H-score > 60) is closely related to the efficacy of platinum-taxane regimens and can be used as an independent predictor of efficacy (12). SLFN11 promoter methylation leads to decreased expression, which is significantly associated with shortened progression-free survival (PFS) and overall survival (OS) in patients with serous ovarian cancer (6), further confirming the key role of SLFN11 expression level in prognosis. In HGSOC samples, the transcription level and protein level of SLFN11 were positively correlated, and high expression level was closely associated with better prognosis of patients (12). The mechanism of action of SLFN11 is related to its function in the DNA damage response (DDR). After DNA damage induction, SLFN11 selectively inhibits the translation of key DDR repair genes (such as ATR and ATM) by mediating tRNA downregulation, thereby impairing the repair capacity of tumor cells (24). In tumor-infiltrating lymphocytes (TILs), the expression level of SLFN11 in non-tumor cells is positively correlated with the number of TILs (12). Analysis of the TCGA HGSOC dataset confirmed that SLFN11 is expressed in macrophages, T cells, and B cell subsets, and is associated with a variety of immune features, including immunogenic cell death features and IFN-γ response features (12). This suggests that SLFN11 not only affects tumor cells themselves, but is also related to the shaping of the anti-tumor immune microenvironment. In a phase II randomized controlled clinical trial of olaparib maintenance therapy, high SLFN11 expression levels were associated with improved prognosis in patients treated with olaparib. Although this association was not completely independent of BRCA mutation status, it suggests that SLFN11 may serve as a supplementary predictive marker in the context of BRCA mutations or for stratification of BRCA wild-type patients, which is worth verifying in larger studies (59). Given that SLFN11 impairs DNA repair by inhibiting the translation of key DDR genes such as ATR, it is theoretically possible that tumor cells with low SLFN11 expression may be more dependent on residual ATR pathway activity for survival, making them particularly sensitive to ATR inhibitors. This theory has been initially supported by clinical studies: a phase II clinical trial (60) conducted in patients with platinum-resistant HGSOC showed that the ATR inhibitor berzosertib combined with gemcitabine significantly prolonged PFS compared with gemcitabine alone (22.9 weeks vs 14.7 weeks, p=0.044). Unfortunately, the study did not evaluate SLFN11 status. Future studies should focus on analyzing whether SLFN11 expression levels (especially low expression) can predict patients’ sensitivity to combined treatment with ATR inhibitors, which will provide an important basis for precision treatment.

### 3.6 Prostate cancer

SLFN11 expression level is a promising predictive biomarker: it not only predicts the benefit of platinum-based chemotherapy in patients with metastatic castration-resistant prostate cancer (mCRPC), but also shows the potential to predict the sensitivity of specific subpopulations (such as RB1 WT AR+) to new targeted therapies such as B7H3-PBD-ADC, providing an important basis for personalized treatment strategies for advanced prostate cancer.

SLFN11 is overexpressed in a significant proportion of advanced prostate cancers, including approximately 45% of metastatic castration-resistant prostate cancer (mCRPC) and 25% of primary prostate cancer (41). Importantly, high SLFN11 expression (greater than the median value of SLFN11 expression is high) was a strong predictor of responsiveness to platinum-based chemotherapy in patients with mCRPC, associated with significantly improved efficacy and longer progression-free survival (PFS) (41). The study also found that SLFN11 expression levels were positively correlated with the efficacy of the antibody-drug conjugate B7H3-PBD-ADC in a metastatic prostate cancer model; in particular, high SLFN11 expression was identified as a key factor in sensitivity to the drug in RB1 wild-type (WT) androgen receptor-positive (AR+) patients (13). The expression of SLFN11 is highly clinically detectable and can be reliably assessed at the mRNA or protein level in tumor tissue or circulating tumor cells (CTCs), with high concordance between the two methods (41).

### 3.7 Ewing sarcoma

SLFN11 is a key molecule in Ewing sarcoma that is directly regulated by the oncogenic driver EWS-FLI1. Its high expression not only has diagnostic and prognostic value, but also is a core biomarker and potential hub for predicting tumor sensitivity to multiple targeted therapy strategies (especially DNA damage response targeted therapy).

In Ewing sarcoma (ES), SLFN11 was shown to be a direct transcriptional target of the core oncogenic driver EWS-FLI1, and its expression is positively regulated by EWS-FLI1 through promoter binding (14). Compared with other pediatric tumors (e.g., neuroblastoma, rhabdomyosarcoma), SLFN11 is significantly overexpressed in ES cell lines, laying the foundation for its use as an ES-specific molecular marker (61). The expression level of SLFN11 is a key determinant of ES sensitivity to a variety of DNA damaging agents. Its high expression is closely associated with tumor sensitivity to topoisomerase I inhibitors (such as SN-38/irinotecan and its nanoliposome form), PARP inhibitors, and trabectedin (14, 61–63). The core mechanism is that SLFN11 can hinder DNA replication fork repair and significantly enhance the replication stress effect induced by these drugs, thereby effectively promoting tumor cell death (61, 62, 64). This mechanism also explains why high SLFN11 expression also predicts the sensitivity of ES to ribonucleotide reductase (RNR) inhibitors (64). Clinical studies have confirmed that ES patients with high SLFN11 expression have a better prognosis (14). Importantly, preclinical models (*in vitro* and *in vivo*) consistently demonstrated that combination therapy with PARP inhibitors and topoisomerase I inhibitors exhibited significant synergistic antitumor activity in ES with high SLFN11 expression (62). The expression level of SLFN11 directly affects the therapeutic effect. Its reduced expression leads to resistance to the above-mentioned DNA damaging agents (61, 63). Crucially, this drug resistance mediated by low SLFN11 expression can be partially reversed by co-application of ATR inhibitors (61, 63), further highlighting the hub status of SLFN11 in the DNA damage response pathway.

The function of SLFN11 is not limited to the DNA damage response. Its expression level was found to directly regulate the sensitivity of ES cells to eltrombopag, a drug that inhibits proliferation through an iron chelation mechanism. Overexpression of SLFN11 enhanced sensitivity, while knockdown of SLFN11 reduced sensitivity (65), demonstrating a role for SLFN11 in a broader therapeutic mechanism.

Notably, although high SLFN11 expression is a strong predictor of sensitivity to DNA damaging agents, some ES cells with high SLFN11 expression still show drug resistance (62). Further studies have shown that such drug resistance is usually not related to the function of SLFN11 itself, but is caused by the impairment of downstream effector pathways (such as apoptosis inhibition, such as BCL-xL overexpression (62). In addition to being a biomarker, AP-1 signaling pathway activated by SLFN11 has been shown to inhibit ES cell growth and downregulate the expression of the oncogene c-Myc (66), suggesting that SLFN11 or its regulatory pathway itself may also be a potential target for therapeutic intervention.

### 3.8 Glioblastoma

In glioblastoma (GBM), the SLFN11 gene is highly expressed, and it promotes GBM progression by negatively regulating the non-classical NFκB signaling pathway. The study used CRISPR/Cas9 technology to knock out SLFN11, and the results showed that knockout significantly inhibited the proliferation and neurosphere formation ability of GBM cells, accompanied by downregulation of the expression of precursor cell/stem cell marker genes (such as NES, SOX2, and CD44), indicating that SLFN11 deficiency weakened tumor stemness. Mechanistically, SLFN11 deficiency directly stimulated the expression of NFκB target genes, including the cell cycle inhibitory protein p21; since upregulation of p21 can block cell cycle progression, this explains the growth inhibition phenotype and confirms the negative regulatory effect of SLFN11 on the NFκB pathway (i.e., SLFN11 deficiency leads to pathway de-inhibition and activation). Furthermore, in a GBM mouse model, SLFN11 deficiency significantly inhibited tumor growth and prolonged survival (15).

### 3.9 Head and neck squamous cell carcinoma

Patients with head and neck squamous cell carcinoma (HNSCC) have significant differences in their responses to cisplatin-based chemoradiotherapy (CRT), and SLFN11 expression levels have been revealed as a key prognostic factor that can predict treatment benefit. Specifically, clinical studies have shown that SLFN11-positive group (SLFN11 positive staining was defined as ≥15% staining of the tumor nuclei) is closely associated with longer progression-free survival; *in vitro* experiments have further confirmed that high SLFN11 expression enhances the sensitivity of cells to platinum drugs (DNA damaging agents), highlighting its potential as a response biomarker (16). Mechanistically, SLFN11 deficiency specifically reduces the sensitivity of cells to DNA-damaging drugs, but has no effect on non-DNA-damaging drugs (such as docetaxel), which emphasizes the central role of SLFN11 in the DNA damage response pathway (16). Interestingly, SLFN11 also has a radiosensitizing effect, and the radiosensitization of DNA-dependent protein kinase (DNA-PK) inhibitors is associated with the expression of SLFN11 mRNA (67).

### 3.10 Colorectal cancer

SLFN11 profoundly affects the response of colorectal cancer (CRC) to DNA-damaging chemotherapy drugs, including irinotecan and platinum (oxaliplatin, cisplatin), by participating in the DNA damage response pathway. Its expression level, especially in the context of KRAS wild-type, has important prognostic value; and its frequent epigenetic silencing (methylation) is one of the key mechanisms leading to chemotherapy resistance and adverse clinical outcomes.

The expression level of SLFN11 is a key determinant regulating the sensitivity of colorectal cancer (CRC) cells to DNA-damaging chemotherapeutic drugs. *In vitro* studies have shown that high expression of SLFN11 can significantly enhance the sensitivity of CRC cells to irinotecan’s active metabolite SN-38, manifested by strong anti-proliferative effects, cell apoptosis and cell cycle arrest, which directly confirms that SLFN11 plays an indispensable role in the DNA damage response pathway induced by irinotecan (17). This effect is also clinically significant in platinum drugs. In CRC patients receiving adjuvant chemotherapy with oxaliplatin, the study revealed an important stratification effect: in KRAS exon 2 wild-type patients, high SLFN11 expression (the final score (0–6) was calculated from the ratio and staining intensity, and a score >4.5 was defined as high expression) was closely associated with significantly prolonged overall survival (OS), indicating a good prognosis; however, in KRAS exon 2 mutant patients, SLFN11 expression levels were not significantly correlated with OS (18). This finding clearly establishes SLFN11 as a potential predictive biomarker for response to oxaliplatin in KRAS wild-type CRC patients. Notably, the expression of SLFN11 itself is significantly affected by epigenetic regulation. About 55.47% of CRC samples have methylation in the promoter region of the SLFN11 gene. This epigenetic silencing event directly leads to a significant decrease in the expression level of SLFN11. Functionally, the loss of expression mediated by SLFN11 methylation weakens the sensitivity of CRC cells to another important platinum drug, cisplatin. More importantly, clinically, the methylation status of SLFN11 is an independent poor prognostic factor, which is clearly associated with patients’ poor 5-year overall survival (OS) and significantly shortened recurrence-free survival (RFS) (68).

### 3.11 Clear cell renal cell carcinoma

SLFN11 is a key tumor promoter and poor prognostic marker for clear cell renal cell carcinoma (ccRCC). Its overexpression (Greater than the median value of SLFN11 expression is high) is an independent prognostic factor and is associated with T stage (T3-T4), distant metastasis (M1), high pathological stage, and death (P < 0.01) (69). SLFN11 is significantly overexpressed at both mRNA and protein levels in ccRCC tissues and cell lines (such as ACHN and 786-O), and promoter hypomethylation may be the reason for its upregulation (69). Functionally, knockdown of SLFN11 can effectively inhibit the proliferation, migration and invasion of ccRCC cells and promote cell apoptosis (19). One of its core cancer-promoting mechanisms is to activate the PI3K/AKT signaling pathway: SLFN11 knockdown inhibits the phosphorylation of this pathway, and this effect can be reversed by the PI3K activator 740Y-P (19).

More importantly, SLFN11 is closely associated with the shaping of the immunosuppressive tumor microenvironment (TME) in ccRCC, which constitutes another key mechanism for its cancer promotion (69). SLFN11 expression is positively correlated with the abundance of various tumor-infiltrating lymphocytes (TILs), including CD4+ T cells, CD8^+^ T cells, macrophages, neutrophils, and dendritic cells. At the same time, it is significantly positively correlated with various immune checkpoint genes (such as CD86, CTLA4, CD244, CD48, CD27, CD40) and key chemokines (such as CXCL5, CXCL10, CXCL11, CXCL13) and their receptors (such as CXCR3, CXCR4, CXCR5, CXCR6, CCR5, CCR6). Functional enrichment analysis (GO/KEGG/GSEA) further confirmed that SLFN11 is involved in immune-related processes such as T cell activation, chemokine signaling pathways, and leukocyte migration. It is worth noting that studies have shown that in ccRCC, chemokines such as CXCL13 (whose receptor CXCR5 is strongly positively correlated with SLFN11) can promote tumor progression by binding to CXCR5 to activate the PI3K/AKT/mTOR pathway, and high expression of CXCR3/4/5/6 is associated with poor overall survival (OS) of patients (70, 71). Therefore, we speculate that SLFN11 may shape an immunosuppressive/pro-tumor TME through regulation (especially inducing specific chemokine networks and immune checkpoint expression), which synergizes with its directly activated PI3K/AKT signaling pathway to jointly drive the aggressive progression and poor prognosis of ccRCC. PPI network analysis: SLFN11 interacts with genes such as SAMHD1 and ETS1 (69). SAMHD1 has been found to play an important role in cell cycle, cancer and innate immunity (72, 73). These interactions may be involved in mediating its regulation of the immune microenvironment and deserve further study in the future.

The clinical significance of SLFN11 needs to be considered in conjunction with the current status of ccRCC treatment. Although the DNA repair-related function of SLFN11 gives it a “beneficial” predictive value in patients treated with DNA-damaging drugs (such as platinum and PARP inhibitors) (14, 18, 40, 41), the first-line treatment of ccRCC mainly relies on anti-angiogenic drugs and immune checkpoint inhibitors (ICIs). In this context, the immunosuppressive microenvironment driven by SLFN11 (manifested by high immune checkpoint expression and specific immune cell composition) may become a key factor affecting the efficacy of treatment. We believe that in ccRCC, this immunomodulatory effect of SLFN11 dominates the cancer-promoting mechanism and may mask the impact of its function in DNA damage response on current mainstream treatment options.

### 3.12 Hepatocellular carcinoma

Recent studies have strongly suggested that SLFN11 is a key regulator in the immune microenvironment of hepatocellular carcinoma (HCC) and shows great potential as a biomarker for predicting the efficacy of immune checkpoint inhibitors (ICI) treatment.

SLFN11 is often downregulated in HCC, and its low expression (Samples with a negative or weak H-score were determined to be the low protein expression group) is significantly associated with poor prognosis of patients (20). Functionally, SLFN11 has been shown to effectively inhibit the proliferation, migration, invasion and metastasis of HCC cells and promote apoptosis. Its molecular mechanism involves interaction with RPS4X, leading to weakened S6 and eIF4E phosphorylation in the ribosome complex, thereby inhibiting the cancer-promoting mTOR signaling pathway. More importantly, recent studies (2024) revealed the central role of SLFN11 in shaping the immune microenvironment of HCC (74). The study found that SLFN11 expression was significantly upregulated in tumor tissues of HCC patients who responded to ICI treatment. In contrast, SLFN11 deficiency promoted the infiltration of immunosuppressive macrophages and aggravated tumor progression. Mechanistic studies have shown that SLFN11 stabilizes RBM10 and promotes NUMB exon 9 skipping by inhibiting TRIM21-mediated RBM10 degradation, a process that is critical for regulating anti-tumor immune responses. It is worth noting that for patients with low SLFN11 expression, the study proposed a potential intervention strategy: blocking the CCL2/CCR2 signaling pathway can effectively enhance their sensitivity to ICI treatment, which provides a new idea for overcoming immunotherapy resistance in such patients (74).

### 3.13 Other

SLFN11 is a predictive biomarker for bladder cancer patients receiving platinum-based chemotherapy, and its expression level can specifically predict chemotherapy response and patient survival outcomes (42). This conclusion is based on a clinical study of 120 cases of bladder cancer: the patients were divided into two groups, the first group (50 cases) were patients with unresectable locally advanced or metastatic bladder cancer who received platinum-containing chemotherapy, and the second group (70 cases) were patients who received surgical resection without chemotherapy. The key findings showed that in the chemotherapy group, the overall survival rate of SLFN11-positive patients (SLFN11 was considered positive when at least 5% of the tumor cells were stained) was significantly better (P=0.012), and SLFN11 expression was positively correlated with the luminal subtype marker GATA3 (p=0.027). In contrast, in the non-chemotherapy group, the overall survival rate of SLFN11-positive patients was worse (P=0.034), which highlights the “predictive” nature of SLFN11-its benefits are only manifested in the context of chemotherapy, probably because high expression of SLFN11 marks the inherent sensitivity of the tumor to DNA damaging agents, but in the absence of chemotherapy, it is associated with an aggressive phenotype.

*In vitro* mechanistic experiments (42) further confirmed the causal role of SLFN11: in bladder cancer cell lines, SLFN11 gene knockout led to resistance to cisplatin, while epigenetic modification drugs (such as 5-azacytidine and entinostat) restored SLFN11 expression and resensitized SLFN11-negative cells to cisplatin and carboplatin. This provides a molecular basis for SLFN11 as a biomarker and suggests that epigenetic therapy can reverse resistance.

Notably, the predictive value of SLFN11 may extend to other cancers. For example, SLFN11 expression is elevated in acute myeloid leukemia (AML) and acute lymphoblastic leukemia (ALL) cells. High expression of SLFN11 is regulated by interferon-JAK signaling and ETS family transcription factors (such as ETS-1 and FLI1) (75); JAK, AKT, ERK, or ETS inhibitors can all downregulate SLFN11. Similarly, in mesothelioma cells, high SLFN11 expression correlated with response to PARP inhibitors, and combination with temozolomide enhanced the sensitivity of cells with low MGMT expression (76), further supporting the broad potential of SLFN11 as a biomarker of DNA damage response (76).

## 4 Potential treatment strategies based on the expression status of SLFN11

### 4.1 Epigenetic re-expression of SLFN11: a combined strategy to enhance chemotherapy efficacy

SLFN11 is an important DNA damage response factor. Its promoter hypermethylation leads to silencing expression, which is a key mechanism for various cancers (such as small cell lung cancer (SCLC)) to develop resistance to platinum and other DNA damaging agents (DDA), and is directly related to the poor prognosis of patients (6). Therefore, reversing SLFN11 silencing through epigenetic drugs and directly restoring its function is an effective way to overcome drug resistance.

DNA demethylating agents (such as decitabine) and histone deacetylase (HDAC) inhibitors (such as FK228) can effectively reverse the abnormal methylation status of the SLFN11 promoter, and FK228 has been shown to upregulate SLFN11 expression in a dose-dependent manner (47, 77, 78). Re-expression of SLFN11 can significantly restore cancer cell sensitivity to DDA: Decitabine significantly enhanced the efficacy of the TROP2-targeting antibody-drug conjugate sacituzumab govitecan by upregulating SLFN11 and TROP2 (a cell surface antigen highly expressed in various epithelial cancers) (78). The HDAC inhibitor FK228 restores sensitivity of SCLC cells to the topoisomerase I inhibitor topotecan (47). More extensive studies have shown that class I HDAC inhibitors can universally induce SLFN11 expression and effectively overcome multiple DDA resistance, but class II HDAC inhibitors have no such effect (77). The EZH1/2 inhibitor valemetostat combined with irinotecan showed efficacy in relapsed SCLC (38), further confirming the clinical translational potential of combining epigenetic drugs with chemotherapy to enhance the therapeutic effect by reactivating SLFN11.

### 4.2 Overcoming SLFN11 deficiency: targeting the DDR pathway to achieve synthetic lethality

SLFN11-deficient tumors rely on the S-G2/M checkpoint to repair DNA damage and survive, leading to DDA resistance (56). Targeting checkpoint kinases such as ATR/CHK1/WEE1 can abrogate this survival pathway.

Preclinical studies have demonstrated the efficacy of DDR inhibitors in overcoming SLFN11 deficiency-associated drug resistance: ATR/CHK1 inhibitors (such as M4344, M6620, and SRA737) have been reported in the clinic to resensitize SLFN11-deficient cells to topoisomerase inhibitors, PARP inhibitors, and cisplatin (33). *In vitro* experiments and PDX models showed that gemcitabine combined with other DDRi (such as ATR inhibitors, WEE1 inhibitors or CHK1 inhibitors) can overcome gemcitabine resistance in SLFN11-deficient cell lines or PDX models (56). Low-dose M1774 showed high synergy with a variety of clinical DDAs, including TOP1 and TOP2 inhibitors, cisplatin, RNA polymerase II inhibitors, and PARP inhibitors (51). M1774 reversed the chemotherapeutic resistance of cancer cells lacking SLFN11 expression to anticancer DDAs. In cell lines or PDX/xenograft models of breast cancer, colon cancer, and SCLC, ATR inhibitors or CHK1 inhibitors combined with chemotherapy regimens (such as TOP1 inhibitors exatecan, lurbinectedin, and PARP inhibitors) showed synergistic effects (50, 79, 80). Notably, ATR inhibition directly reverses SLFN11 deficiency-associated resistance to DNA-damaging agents, pyrrolobenzodiazepine dimer (57) and PARP inhibitors (7), by blocking the S phase checkpoint. This strategy has shown positive clinical translational signals: gemcitabine combined with an ATR inhibitor showed efficacy in a phase II trial for high-grade serous ovarian cancer (60); an ATR inhibitor combined with a PARP inhibitor/lurbinectedin (an alkylating agent/DNA damaging agent) showed synergistic effects in SCLC cell lines (50, 79). Together, these results highlight targeting the DDR pathway as a powerful therapeutic prospect to overcome SLFN11 loss-associated drug resistance and achieve synthetic lethality.

## 5 Challenges and future prospects

Future research directions based on SLFN11 should focus on the selection of evaluation methods, dynamic monitoring technology, optimization of combination therapy, elucidation of molecular mechanisms, cross-cancer validation, and regulation of the immune microenvironment. By focusing these research directions, it not only addresses the limitations of existing treatments (such as drug resistance and heterogeneity),but also provides an actionable path for clinical translation. The following suggestions are directly related to the core functions of SLFN11 and the reported treatment strategies, which contribute to promoting its transition from a biomarker to a therapeutic target.

### 5.1 Detection methods for SLFN11

SLFN11 can predict the efficacy of DNA-targeted drugs in various tumors, and this effect has been confirmed by immunohistochemistry (IHC) in multiple previous studies (12, 20, 36, 37). Additionally, we analyzed two datasets, GSE37751 and GSE29013, from the Gene Expression Omnibus (GEO) database. When only analyzing breast cancer patients who received chemotherapy (n = 34), patients with high SLFN11 expression showed a significant benefit in overall survival (OS) (p = 0.048). Similarly, in a dataset of 110 ovarian cancer patients who received cisplatin chemotherapy, high SLFN11 expression showed a trend associated with longer OS (p = 0.053). However, some studies suggest that the expression level of SLFN11 obtained by tissue RNA-seq may be overestimated in certain tumor tissues. In addition to tumor cells, there are other non-tumor cells (such as immune cells) in tumor tissues, and SLFN11 is also expressed, or even strongly expressed, in these non-tumor cells (12, 81). The study compared the RNA-seq data of SLFN11 in the TCGA database with the IHC staining of clinicopathological tissue specimens, and emphasized the importance of using IHC rather than tissue RNA-seq to evaluate the expression of SLFN11 in patient samples (81). In a study on high - grade serous ovarian cancer, they separately investigated the IHC semi - quantitative H - scores of SLFN11 in tumor and non - tumor cells, emphasizing the hypothesis that cancer - expressed SLFN11 is directly related to the sensitivity of tumor cells to DNA - damaging agents such as platinum. Moreover, they believed that the overall SLFN11 H - score is a more powerful prognostic biomarker compared to the separately measured cancer or non - cancer SLFN11 (12). A study on the prognostic role of SLFN11 in bladder cancer only evaluated the expression score of SLFN11 in tumor cells and found that SLFN11 was associated with better overall survival (OS) in patients receiving platinum - based chemotherapy (p = 0.012) (42). In some tissues (such as breast and pancreatic tissues), there are significant differences in the distribution of TCGA and IHC between normal and tumor tissues (81). For different types of cancers, there are some differences in the selection of SLFN11 detection methods (IHC or RNA - seq) and evaluation regions (non - tumor cells, tumor cells, or overall). Further research is required in different cancers to screen and evaluate specific evaluation strategies.

### 5.2 Development and validation of a dynamic monitoring technology for SLFN11 expression

Research has shown that SLFN11 undergoes dynamic changes during the treatment process (52), and its expression status can affect the efficacy of DNA-targeted drugs. Therefore, dynamic detection of SLFN11 expression is particularly important for the precise treatment of cancer patients. Non-invasive detection methods based on liquid biopsy (such as circulating tumor cells) should be developed to monitor the dynamic changes in SLFN11 expression during the treatment in real time, especially the downward trend of SLFN11 expression after chemotherapy or treatment with PARP inhibitors. Dynamic monitoring data can be used to guide the timing of combination therapy with ATR inhibitors, ATM inhibitors, CHK1 inhibitors, WEE1 inhibitors or EZH2 inhibitors. For example, timely intervention when the expression of SLFN11 decreases can be carried out to overcome drug resistance. In addition, given the challenges in obtaining sufficient tumor tissues from non - small cell lung cancer, liquid biopsy should be regarded as an important tool in research and treatment. In several preclinical studies using cell lines and patient - derived xenograft models, the expression of SLFN11 strongly predicted the response to cisplatin and PARP inhibitors (8, 9). Therefore, the dynamic detection of SLFN11 in circulating tumor cells shows special potential. For example, screening out the SLCC population sensitive to cisplatin and PARP inhibitors through dynamic monitoring of liquid biopsy and timely intervention when SLFN11 expression decreases all require more prospective studies for verification.

### 5.3 Optimization of combination therapies based on SLFN11 status

High expression of SLFN11 is associated with the sensitivity of tumor cells to DNA-targeted drugs, while low expression of SLFN11 is associated with the resistance of tumor cells to DNA-targeted drugs. It is of potential value to explore combined treatment options based on the expression status of SLFN11 (**Table 3**). The combination of the PARP inhibitor talazoparib and the immune checkpoint inhibitor atezolizumab as maintenance therapy significantly improved the progression-free survival (PFS) of patients with SLFN11-positive small cell lung cancer (SCLC) (39). In tumor-infiltrating lymphocytes (TILs), the expression level of SLFN11 in non-tumor cells was positively correlated with the number of TILs (12). Breast cancer samples with high SLFN11 expression were accompanied by enhanced immune response characteristics, including T cell infiltration and high expression of immune checkpoints (such as PD-L1) (56, 58, 82). The results of a phase II clinical study comparing the ATR inhibitor berzosertib in combination with gemcitabine versus gemcitabine alone for the treatment of platinum-resistant high-grade serous ovarian cancer showed that the combination therapy group significantly prolonged progression-free survival (PFS) (22.9 weeks vs 14.7 weeks, p = 0.044) (60). For tumors with high SLFN11 expression, exploring the synergistic effect and mechanism of action of immune checkpoint inhibitors combined with PARP inhibitors or chemotherapy has potential clinical significance. For tumors with low SLFN11 expression, systematically evaluate the efficacy of the combination regimens of ATR inhibitors, ATM inhibitors, CHK1 inhibitors, and WEE1 inhibitors with standard chemotherapy in different cancers.

**Table 3:** Potential treatment strategies based on the expression status of SLFN11.

Strategy classification	Specific methods	Applicable scenarios	Mechanism of action
Epigenetic regulation	HDAC inhibitors (FK228) or demethylation agents (5-AZA/DAC) induce SLFN11 expression	SLFN11 low expression tumor	Reverse promoter methylation or histone modification to restore the expression of SLFN11
Combination therapy (high expression of SLFN11)	1. Combination of PARP inhibitors and immune checkpoint inhibitors (small cell lung cancer, breast cancer); 2. Combination of PARP inhibitors and topoisomerase inhibitors (Ewing sarcoma)	1. SCLC, breast cancer 2. Ewing sarcoma	1. High expression of SLFN11 in tumors with enhanced immune response characteristics, Enhance DNA damage 2. Enhance DNA damage
Combination therapy (low expression of SLFN11)	1. ATR inhibitor + chemotherapy, 2. ATR inhibitor + PARP inhibitor, 3. EZH2 inhibitor + chemotherapy, 4. ATR inhibitor + lurbinectedin, 5. CCL2/CCR2 inhibitor + immune checkpoint inhibitor, 6. ATM inhibitors + chemotherapy	1. Breast cancer 2. SCLC 3. SCLC 4. SCLC 5. Hepatocellular carcinoma 6. Esophageal cancer	Inhibit the replication stress checkpoint to overcome the drug resistance caused by SLFN11 deficiency.
Targeting the functional module of SLFN11	Kinase inhibitors regulate the phosphorylation sites (such as S753) of SLFN11.	–	Regulate the conformation of SLFN11 and its binding ability to ssDNA.
Dynamic monitoring and precise intervention	Liquid biopsy (CTC/ctDNA) monitors the dynamic expression of SLFN11 to guide the timing of combination therapy with ATR/ATM/EZH2 inhibitors.	Tumors that progress after chemotherapy or treatment with PARP inhibitors	Adjust the treatment strategy in real - time to prevent drug resistance caused by the down - regulation of SLFN11.

Screen specific epigenetic drugs (such as HDAC inhibitors or low - toxicity demethylating agents) targeting SLFN11 promoter methylation or histone modification, and evaluate the differences in their efficacy among different cancer types. In tumors with low SLFN11 expression, such as ovarian cancer, breast cancer, colorectal cancer, small - cell lung cancer, and bladder cancer, combine chemotherapy with HDAC inhibitors or demethylating agents to verify whether they can enhance chemotherapy sensitivity by upregulating SLFN11.

### 5.4 In - depth analysis and targeted intervention of the molecular mechanism of SLFN11

Analyze the interaction mechanisms of SLFN11 with RPA1, ssDNA, DDB1 of CUL4^CDT2^ E3 ubiquitin ligase, and develop small - molecule drugs to mimic or block its functions. Explore the regulatory network of SLFN11 phosphorylation sites (such as S753) and design kinase inhibitors to modulate its conformation and activity. In glioblastoma, target the interaction between SLFN11 and the NF - κB pathway and verify whether it can reverse the characteristics of tumor stem cells.

### 5.5 Cross-cancer clinical validation and biomarker stratification

Establish a multi - center cohort study and enroll patients with pan - cancer types (such as ovarian cancer, small - cell lung cancer (SCLC), and triple - negative breast cancer (TNBC)). Stratify patients based on the expression level of SLFN11 (using immunohistochemistry (IHC) or next - generation sequencing (NGS)) and evaluate its predictive value for different DNA - targeting drugs (such as PARP inhibitors, platinum - based drugs, and TOP inhibitors). In BRCA wild - type ovarian cancer, determine whether SLFN11 can serve as an independent predictive marker for the efficacy of olaparib to compensate for the limitations of homologous recombination deficiency (HRD) testing.

### 5.6 The interaction between SLFN11 and the tumor microenvironment

Investigate how SLFN11 affects the response to immunotherapy by regulating immune cell infiltration (such as T cells and macrophages) or cytokine secretion (such as the CCL2/CCR2 axis), especially in hepatocellular carcinoma (74) and ovarian cancer (12). In HCC with low SLFN11 expression, combine CCR2 inhibitors with PD - 1 inhibitors to verify whether it can reverse the immunosuppressive microenvironment (74).

## 6 Discussion

SLFN11 has emerged as a pivotal biomarker and potential therapeutic modulator in the era of precision oncology. This review consolidates compelling evidence demonstrating that SLFN11 expression strongly correlates with increased sensitivity to a wide array of DNA-damaging agents (DDAs), including platinum compounds, topoisomerase inhibitors, and PARP inhibitors across diverse cancer types (7, 8, 11). The mechanistic underpinnings of this sensitivity—ranging from replication fork arrest and tRNA cleavage to inhibition of homologous recombination via RPA1 destabilization—are unique and position SLFN11 as a functional gatekeeper of DNA damage response (DDR) (22, 32). Furthermore, the modulation of SLFN11 through epigenetic silencing, post-translational modifications, and transcriptional regulation provides clinically actionable targets for reversing drug resistance.

One of the most striking findings across cancer types is the context-dependent role of SLFN11 in influencing prognosis and therapy response. In cancers such as ovarian, breast, gastric, and small cell lung cancer (SCLC), high SLFN11 expression consistently predicts better outcomes in patients treated with DNA-targeting chemotherapy (10–12, 37, 44). In Ewing sarcoma, SLFN11 is transcriptionally activated by the EWS-FLI1 oncogene and is required for sensitivity to PARP and topoisomerase I inhibitors (14). These data support its role as a lineage-influenced, mechanistically relevant biomarker.

Despite its promise, the utility of SLFN11 as a universal biomarker faces several challenges. A key limitation is the dynamic and heterogeneous expression of SLFN11 both within and between tumors, which can fluctuate during treatment (9, 52). This necessitates the development of real-time, non-invasive monitoring technologies, such as liquid biopsy-based assays using circulating tumor cells (CTCs) (52). Moreover, there is no standardized detection method—while RNA-seq data provide transcriptional snapshots, protein-level evaluation via immunohistochemistry (IHC) may offer a more accurate reflection of functional SLFN11 expression, especially given its expression in immune and stromal cells (12, 81).

Therapeutically, SLFN11-deficient tumors often exhibit intrinsic resistance to DDAs. However, this resistance is not insurmountable. Multiple preclinical studies, including those in SCLC, breast, and colorectal cancers, demonstrate that SLFN11 loss can be overcome by targeting compensatory DDR pathways, particularly ATR, CHK1, and WEE1 (7, 56, 79, 80). This introduces a synthetic lethality-based rationale for combination regimens in SLFN11-low or silenced tumors. Epigenetic drugs, such as HDAC inhibitors and demethylating agents, have shown efficacy in reactivating SLFN11 expression, thereby restoring chemosensitivity (47, 77). These findings underscore the therapeutic flexibility of SLFN11 as both a predictive marker and a targetable resistance mechanism.

Additionally, SLFN11 has emerging significance in shaping the tumor immune microenvironment (TME). Its positive correlation with immune cell infiltration and immune checkpoint expression in several tumor types—most notably in gastric cancer, breast cancer, and hepatocellular carcinoma—suggests an immunomodulatory role that may synergize with immune checkpoint inhibitors (12, 43, 74). Early clinical evidence from SCLC patients receiving PARP inhibitors in combination with ICIs supports this hypothesis, although further prospective trials are needed to validate such approaches (39).

Moving forward, several research directions are warranted. First, pan-cancer prospective clinical trials should evaluate the predictive power of SLFN11-guided therapies, especially in patients without BRCA mutations or homologous recombination deficiency. Second, investigations into post-translational regulation (e.g., S753 phosphorylation) of SLFN11 activity may yield new therapeutic levers (28). Third, integrative studies that stratify patients based on SLFN11 expression alongside other biomarkers (e.g., ATM, EMT status, TIL density) may refine response prediction models (9, 12).

In conclusion, SLFN11 represents a paradigm-shifting biomarker at the intersection of DNA damage response, epigenetics, and immunology. Its integration into clinical oncology not only promises to optimize treatment efficacy and reduce unnecessary toxicity but also offers new avenues for therapeutic innovation, especially in drug-resistant and biomarker-poor cancers. With further validation and clinical translation, SLFN11 has the potential to evolve from a predictive biomarker into a central node of personalized cancer therapy.
