# Correction: SLFN11: a pan-cancer biomarker for DNA-targeted drugs sensitivity and therapeutic strategy guidance

**DOI:** 10.3389/fonc.2026.1779304

**Published:** 2026-02-04

**Authors:** Kunzhong Zhou, Yuewen Li, Weifang Wang, Yilin Chen, Bingyan Qian, Yiteng Liang, Hongmei Li, Ruiting Xu, Li Zhuang

**Affiliations:** Department of Rehabilitation and Palliative Medicine, The Third Affiliated Hospital of Kunming Medical University, Yunnan Cancer Hospital, Peking University Cancer Hospital Yunnan, Kunming, China

**Keywords:** Schlafen11 (SLFN11), DNA damaging agents, PARP inhibitors, pan-cancer, DNA damage repair mechanisms, epigenetics, biomarkers

Affiliation “Department of Rehabilitation and Palliative Medicine, The Third Affiliated Hospital of Kunming Medical University, Yunnan Cancer Hospital, Peking University Cancer Hospital Yunnan, Kunming, China” was erroneously given as “Department of Rehabilitation and Palliative Medicine, The Third Affiliated Hospital of Kunming Medical University, Kunming, China”.

In the published article, there was a mistake in the **Funding** statement. The **Funding** statement for the National Natural Science Foundation of China was displayed as “8226110412, 82460540”. The correct statement is “the National Natural Science Foundation of China (82260589, 82460540).’’

The funder the Multidisciplinary Talent Collaboration Platform for Tumor Support and Rehabilitation, 5222 to Li Zhuang was erroneously omitted.

The funder the Xishan District Talent Work Innovation Project: Construction of a Standardized Cancer Pain Diagnosis and Treatment Talent Collaboration Mechanism, 6015 to Li Zhuang was erroneously omitted.

The correct funding statement reads:

The author(s) declare that financial support was received for the research and/or publication of this article. This review was funded by the Translational Application Research Team Project of Precision Treatment and Diagnosis Strategies for Lung Cancer of Kunming Medical University (ZC2024XKTDPY08), the Special Project for Medical and Health Talents of the “Xingdian Talent Support Program” (CZ0096-901895), the National Natural Science Foundation of China (82260589, 82460540), the Multidisciplinary Talent Collaboration Platform for Tumor Support and Rehabilitation (5222), and the Xishan District Talent Work Innovation Project: Construction of a Standardized Cancer Pain Diagnosis and Treatment Talent Collaboration Mechanism (6015). The funders had no role in the study design, data collection, analysis, decision to publish, or preparation of the manuscript.

The original version of this article has been updated.

